# The MAPKKK Gene Family in *Gossypium raimondii*: Genome-Wide Identification, Classification and Expression Analysis

**DOI:** 10.3390/ijms140918740

**Published:** 2013-09-11

**Authors:** Zujun Yin, Junjuan Wang, Delong Wang, Weili Fan, Shuai Wang, Wuwei Ye

**Affiliations:** State Key Laboratory of Cotton Biology, Institute of Cotton Research of Chinese Academy of Agricultural Sciences, Anyang 455000, Henan, China; E-Mails: zujuny@163.com (Z.Y.); wangjj@cricaas.com.cn (J.W.); wangdl@cricaas.com.cn (D.W.); hai-19@163.com (W.F.); wangshuai_19871201@163.com (S.W.)

**Keywords:** cotton, ovule, MAPK cascade, MAPKKK, gene family

## Abstract

Mitogen-activated protein kinase (MAPK) cascades are conserved signal transduction pathways in all eukaryotic organisms. MAPKKKs (MAPK kinase kinases) operate at the top levels of these cascades. Recently, this family of genes has been systematically investigated in Arabidopsis, rice and maize, but has not yet been characterized in cotton. In this study, we identified 78 putative MAPKKK genes in the genome of the diploid cotton, *Gossypium raimondii*. They were classified into three subfamilies, of which 12 were ZIK, 22 were MEKK and 44 were Raf. The ZIK and MEKK genes displayed a scattered genomic distribution across 11 of the 13 chromosomes, whereas Raf genes were distributed across the entire genome. Their conserved patterns observed for introns and additional domains were consistent with the evolutionary relationships inferred from the phylogenetic analysis within subfamily. Transcriptome sequencing data were used to investigate their transcript profiles in mature leaves, 0 day and 3 days post-anthesis (DPA) ovules. Sixty MAPKKK genes were expressed, of which 41 were strongly expressed in mature leaves. Twelve MAPKKK genes were more highly expressed in 3-DPA ovules than in 0-DPA ovules. Our results provide a foundation for future evolutionary and functional characterizations of MAPKKK genes in cotton and probably other Gossypium plants.

## 1. Introduction

Mitogen-activated protein kinase (MAPK) cascades are evolutionarily conserved signal transduction modules that are found in all eukaryotes [1]. In plants, signaling through the MAPK cascade can lead to diverse cellular activities, including cell division and differentiation, responses to abiotic and biotic stresses and programmed cell death [2,3]. The cascades are composed of three protein kinase modules: MAPK kinase kinase (MAPKKKs/MEKKs), MAPK kinase (MAPKKs/MKKs) and MAPK (MAPKs/MPKs) [1]. They are sequentially activated through phosphorylation by their upstream components. Upstream signals activate the MAPKKKs, which in turn activate the MAPKKs. They result in the activation of the specific MAP kinases. Eventually, the activated MAPK phosphorylate various transcription factors and other signaling components that modulate the expression of downstream genes [4]. The phosphorylation of proteins by MAPKs can affect many aspects of their function, including protein stability, cellular localization, DNA binding, protein-protein interactions and the regulation of other post-translational modifications [5,6]. In addition to phosphorylating target molecules, there is evidence that MAPK cascade components can also non-enzymatically regulate transcription [7].

In plants, MAPKKKs contain long *N*- or *C*-terminal regions compared to MAPKs and MAPKKs [4]. This family has been clustered into three groups based on the sequence in their kinase catalytic domain: the Raf-like family, the MEKK-like family and the ZIK-like family [8]. In Arabidopsis, the well-studied Raf-like MAPKKK genes include the *Constitutive Triple Response1* (*CTR1*) and *Enhanced Disease Resistance1* (*EDR1*) genes [9,10]. CTR1 was found to inhibit MKK9-MPK3/MPK6 activation during ethylene signaling and probably acts as an unconventional MAPKKK [11,12]. *EDR1* encodes a *CTR1*-like kinase and acts as a negative regulator of disease resistance and ethylene-induced senescence [13]. Two rice Raf-like MAPKKKs, named *Accelerated Cell Death and Resistance 1* (*ACDR1*) and *Drought-hypersensitive Mutant 1* (*DSM1*), have been reported to positively regulate fungal disease and drought resistance, respectively [14,15]. Rice Increased Leaf Angle 1 (ILA1) is a key factor regulating mechanical tissue formation at the leaf lamina joint [16]. Tobacco MAPKKK *Nicotiana Protein Kinase 1* (*NPK1*) and Arabidopsis *NPK1-related protein kinase 1* (*ANP*) are found in the equatorial region of phragmoplasts and are involved in cytoskeletal regulation [17]. The *MEKK1* gene, one of the first MAPKKKs to be characterized in Arabidopsis, is involved in pathogen defense and abiotic stress responses [18,19]. It has become evident that the MEKK1-MKK1/2-MPK4 pathway is a central regulator of reactive oxygen species (ROS) metabolism [20]. In tomato, MAPKKKα and MAPKKKɛ act as signaling molecules that positively regulate cell death networks associated with plant immunity [21,22]. Accumulated functional characterization of MAPKKKs has highlighted its diverse function in a developmental, tissue and signal-dependent context.

Recently, MAPKKK gene family has been systematic investigated in Arabidopsis, rice and maize. The Arabidopsis genome contains approximately 80 MAPKKK genes, which include 48 Raf kinases, 21 MEKK kinases and 11 ZIK kinases [8,23]. The rice genome contains 75 MAPKKK genes [24]. Half of them were present in the first three chromosomes. Seventy four MAPKKKs were identified in the sequenced maize genome. The expression profiles of 57 genes were examined in different organs using microarray data [25].

Cotton belongs to the genus *Gossypium*, which consists of diploid and tetraploid species, and has originated from a common ancestor approximately 5–10 million years ago. In scientific research, cotton serves as an excellent model system for studying polyploidization, cell elongation and cell wall biosynthesis. Little is known, however, about the identification and characterization of the MAPKKK gene family in cotton. *Gossypium raimondii* is a diploid cotton. Its progenitor is the putative contributor of the D subgenome to the economically important fiber-producing cotton species *G. hirsutum* and *G. barbadense* [26]. Recently, the *G. raimondii* genome was sequenced, which made it possible to identify all the MAPKKK genes in this species for the first time [27]. In this study, 78 MAPKKK genes were identified from the *G. raimondii* genome. Detailed information on their genomic structures, chromosomal locations and phylogenetic trees is provided. Their transcript profiles in leaves and at the 0 day post-anthesis (DPA) and 3-DPA ovule developmental stages were further investigated using transcriptome sequencing data. Our results provide the basis for future research on their evolutionary mechanisms and the signaling pathways mediated by MAPKKKs in cotton.

## 2. Results and Discussion

### 2.1. Genome-Wide Identification of the MAPKKK Family in *G. raimondii*

MAPK cascades are evolutionarily conserved signaling modules in eukaryotes, such as animals, yeasts and plants. Based on the high degree of sequence conservation, putative orthologs of MAPK cascade members was identified by sequence comparison and signature motif searches [24]. In order to identify MAPKKK genes in *G. raimondii*, MAPKKK protein sequences from Arabidopsis and rice were used as queries in a BLAST search of the publically available *G. raimondii* sequence database. After extensive analysis, a total of 78 genes were defined as *G. raimondii* MAPKKKs (Table 1). During screening of the potential MAPKKKs, the conserved protein domains in their sequences were analyzed using the PROSITE program. All of them contained a serine/threonine protein kinase active site (PS00108), a protein kinase domain (PS50011) and a protein kinase ATP-binding region (PS00107). These characteristic features suggested that they were members of MAPK cascade gene family.

Based on Arabidopsis MAPKKK nomenclature suggestions [28], each gene was named with a two-letter code corresponding to *G. raimondii* (Gr). GrMAPKKKs were numbered from 1 to 78 according to the BLASTP search output from top to bottom (Table 1). The open reading frame (ORF) lengths of the GrMAPKKK genes ranged from 885 bp (*GrMAPKKK2*) to 4200 bp (*GrMAPKKK14*). Their protein sequences contained 294–1399 amino acids (aa), with the majority (84.62%) containing 350–900 aa. The molecular weight (*M*w) of these proteins ranged from 33.68 kDa (GrMAPKKK2) to 154.46 kDa (GrMAPKKK14). The theoretical isoelectric point (pI) ranged from 4.96 (GrMAPKKK5) to 9.29 (GrMAPKKK21), with an average of about 6.75. Protein subcellular localization prediction is an essential step for understanding protein function and its pattern of interactions in protein networks [29]. Most of GrMAPKKK proteins were predicted to be located in the nucleus and the cytoplasm (Table 1). Five GrMAPKKK proteins (GrMAPKKK38, GrMAPKKK45, GrMAPKKK48, GrMAPKKK55 and GrMAPKKK61) were predicted to be located in the mitochondria. Two GrMAPKKK proteins (GrMAPKKK29 and GrMAPKKK44) were predicted to be located in the chloroplast. At present, the subcellular localization of most known MAPKKK proteins remains to be experimentally determined. In rice, *DSM1* is a Raf-like MAPKKK gene functioning as an early signaling component in regulating responses to drought stress by regulating scavenging of ROS [15]. Its protein is confirmed to be located in nucleus by transient expression analysis. Another Raf-like MAPKKK protein ILA1 is predominantly resident in the nucleus and expressed in the vascular bundles of leaf lamina joints [16]. Detailed information about MAPKKK proteins in mitochondria or chloroplast is less in plant kingdom recently. Takabatake *et al.* [30] demonstrated that MAPK cascade could transduce the cell death signal to mitochondria for N gene-dependent cell death through operating downstream of heat shock protein 90 (HSP90) in tobacco.

### 2.2. Phylogenetic Analysis and Genomic Distribution of GrMAPKKKs

**Figure 1:**
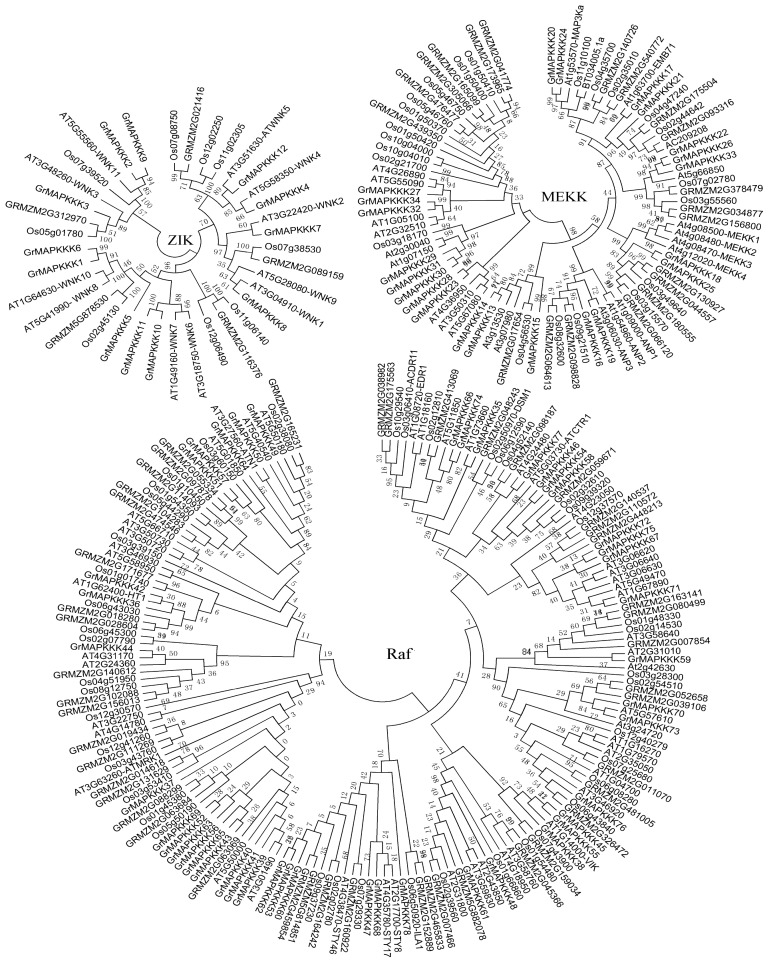
Phylogenetic trees of the MAPKKK family genes in Arabidopsis, rice, maize and *G. raimondii*. The unrooted tree was generated by the MEGA v5 program using the neighbor-joining method. Bootstrap values from 1000 replicates are indicated at each branch. The phylogenetic trees were constructed based on the full-length protein sequences of the MAPKKKs. At, *Arabidopsis thaliana*; Os, *Oryza sativa*; GRMZM, gene model IDs from the Maize Genome Sequencing Project.

MAPKKK genes act at the highest level of the MAPK cascades and form the largest group of components in the MAPK cascade pathway. To investigate the molecular evolution and phylogenetic relationships between different MAPKKK family members, the MAPKKK proteins in Arabidopsis, rice, maize and *G. raimondii* were aligned by ClustalW and analyzed using MEGA v5. Using full-length protein sequences of the MAPKKKs, phylogenetic trees were constructed with the neighbor-joining (NJ) method and the minimal evolution (ME) method. The two methods produced the same topologies, even at the deep nodes. As shown in Figure 1, the phylogenetic trees indicate that the GrMAPKKK genes were placed into three categories, which were based on their sequence similarity with orthologs in other plants. The three categories were ZIK, MEKK and Raf. The ZIK subfamily consists of the fewest number of MAPKKK genes. Twelve GrMAPKKKs belonged to this group. In Arabidopsis, rice and maize, 11 AtMAPKKKs, 10 OsMAPKKKs and 6 ZmMAPKKKs were grouped in this subfamily [24,28]. The MEKK subfamily consists of 22 GrMAPKKK genes in *G. raimondii*. The remaining 44 GrMAPKKK genes belong to the Raf group, which is the largest subfamily of MAPKKKs (Figure 1).

**Figure 2:**
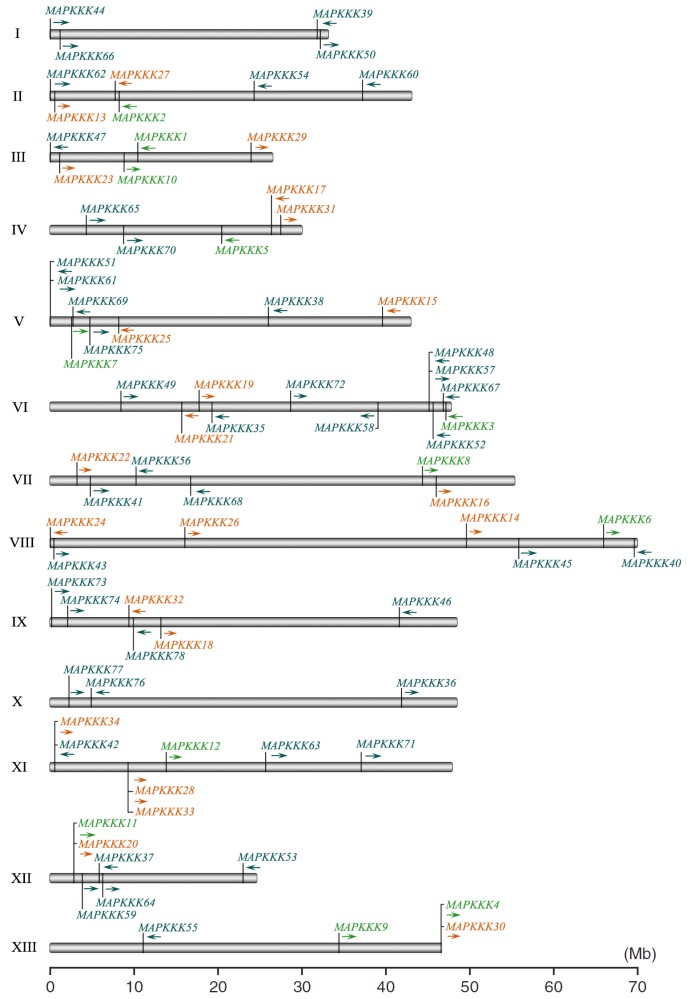
Chromosomal locations of MAPKKK genes in the *G. raimondii* genome. The ZIK, MEKK and Raf subfamily genes are separately indicated with green, orange and blue, respectively. The arrows indicate the rightward or leftward direction of transcription.

The complete genome sequence gave an overview of the chromosomal distribution of GrMAPKKK genes. These important signaling components were mapped on all 13 chromosomes of *G. raimondii* (Figure 2). Chromosome VI carried 11 divergent GrMAPKKK genes and chromosome X contained 3 GrMAPKKK genes. It is thought that gene families can arise through tandem amplification, resulting in a clustered occurrence. They can also arise through segmental duplication of chromosomal regions, resulting in a scattered occurrence of family members [31]. In the *G. raimondii* genome, both the ZIK genes and the MEKK genes were found to be located on chromosomes: II, III, IV, V, VI, VII, VIII, XI, XII and XIII. Although there are many ZIK or MEKK paralogs that have high levels of sequence similarity, nevertheless, they showed a scattered genomic distribution across 11 of the 13 chromosomes. Recent studies have shown that the *G. raimondii* genome has undergone at least two rounds of genome-wide duplication, 2355 syntenic blocks have been identified and 39 triplicated regions [27]. The scattered genomic distribution pattern of the two subfamily genes probably reflects a series of whole genome, chromosomal and large segmental duplication events that typify the *G. raimondii* genome. Forty-four Raf subfamily members were found on all 13 chromosomes. All of them were also randomly distributed across the genome, apart from *GrMAPKKK48*, *GrMAPKKK51*, *GrMAPKKK57* and *GrMAPKKK61. GrMAPKKK48* and *GrMAPKKK57* were found to be physically located near to each other on chromosome VI. *GrMAPKKK51* and *GrMAPKKK61* formed a cluster and were located on chromosome V. The two clusters consisted of two Raf gene pairs: *GrMAPKKK48*/*GrMAPKKK61* and *GrMAPKKK51*/*GrMAPKKK57*. This pattern probably results from translocation and insertion events between the two chromosomes. Twelve GrMAPKKK paralogous gene pairs were found after phylogenic analysis. They were in the same clade of the phylogenetic tree, which suggested that the GrMAPKKK gene family may have undergone multiple duplications during its evolutionary history. It has been reported that 75 OsMAPKKKs are distributed on all 12 chromosomes of rice and half of them are present on the first three chromosomes [24]. In contrast, the 74 ZmMAPKKKs are distributed on all 10 chromosomes of maize without physical accumulation [25]. The scattered distribution of *G. raimondii* MAPKKK genes suggested that recent duplication events have occurred in this gene family.

### 2.3. Gene Structural Organization and Domain Analysis of GrMAPKKKs

**Figure 3:**
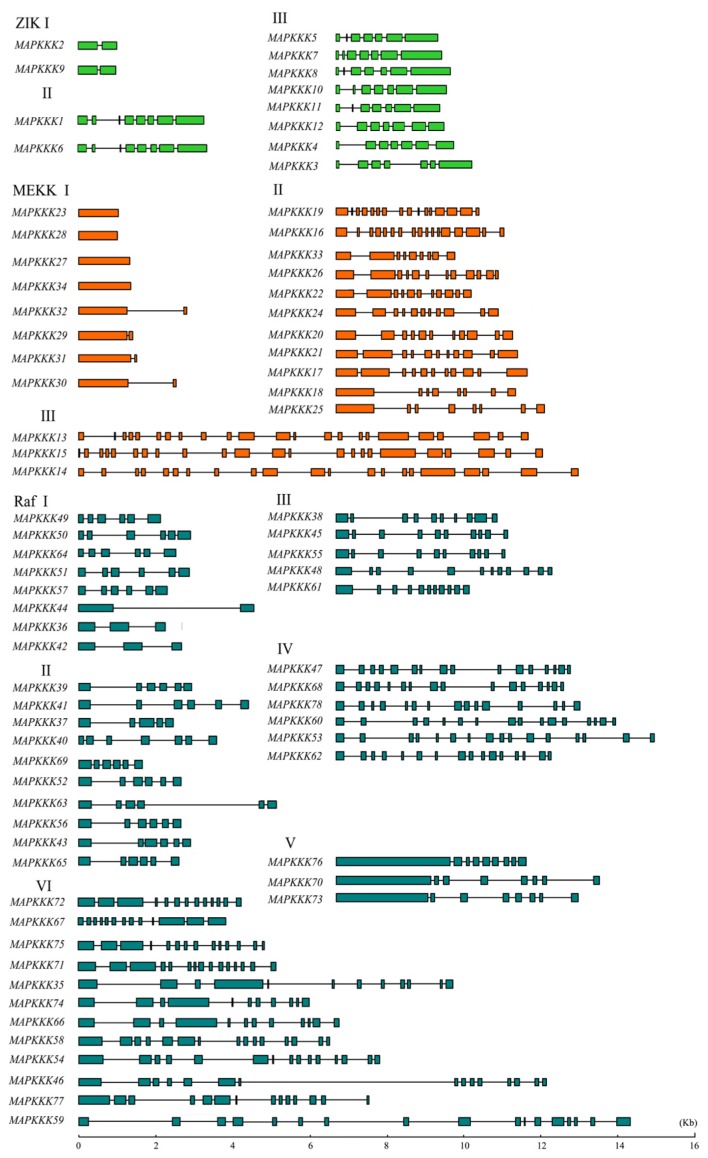
Intron and exon organization of MAPKKK family genes in *G. raimondii*. Introns and exons are represented by black lines and colored boxes, respectively. *MAPKKK* genes have been grouped according to phylogenetic classification.

Analysis of the exon-intron junction patterns can provide additional insights into the evolution of gene families. In order to obtain some insight into the gene structures of GrMAPKKK family genes, their exon/intron organizations were analyzed. The differences in gene structure among GrMAPKKKs were significant. As shown in Figure 3, the GrMAPKKKs had exons varying from one (*GrMAPKKK23*, *GrMAPKKK27*, *GrMAPKKK28* and *GrMAPKKK34*) to 23 (*GrMAPKKK13* and *GrMAPKKK15*). A majority of the GrMAPKKKs (83%) had more than 3 exons. ZIK subfamily members can be divided into three groups, according to their exon/intron structures. The *GrMAPKKK2* and *GrMAPKKK9* gene pairs had 2 exons and the *GrMAPKKK1* and *GrMAPKKK6* gene pairs had 8 exons. The remaining ZIK genes had 7 exons. The gene structures of the ZIK subfamily members were less divergent than the MEKK and Raf subfamilies. This suggested that the genes in this family had preserved a relatively constant exon-intron composition during the evolution of the *G. raimondii* genome. Exons are considered to be under a high selection pressure compared to introns. However, some losses or gains of exons were identified during the evolution of the MEKK family genes. For example, there was a small segment at the 3′ terminal of *GrMAPKKK29*-32, but not in *GrMAPKKK23*, *GrMAPKKK27*, *GrMAPKKK28* and *GrMAPKKK34*, although they were within the same phylogenetic group. The Raf subfamily could be divided into six groups. The first five groups had conserved structural patterns, while group VI possessed a complex distribution of exons and introns. The intron numbers for the Raf subfamily genes ranged from two (*GrMAPKKK36* and *GrMAPKKK42*) to 16 (*GrMAPKKK59*). Despite differences in the length of particular introns, it was clear that the exon structural pattern was well conserved between close paralogs, such as *GrMAPKKK46* and *GrMAPKKK54. GrMAPKKK35*, *GrMAPKKK66* and *GrMAPKKK74* were in the same phylogenetic group. When their structural patterns were compared, we found that there was a missing exon in the middle of *GrMAPKKK66* sequence. This indicated that the exon/intron structures of each gene cluster originated from tandem or segmental duplication events and tended to share a similar structural organization, but with evolutionary tiny difference.

**Figure 4:**
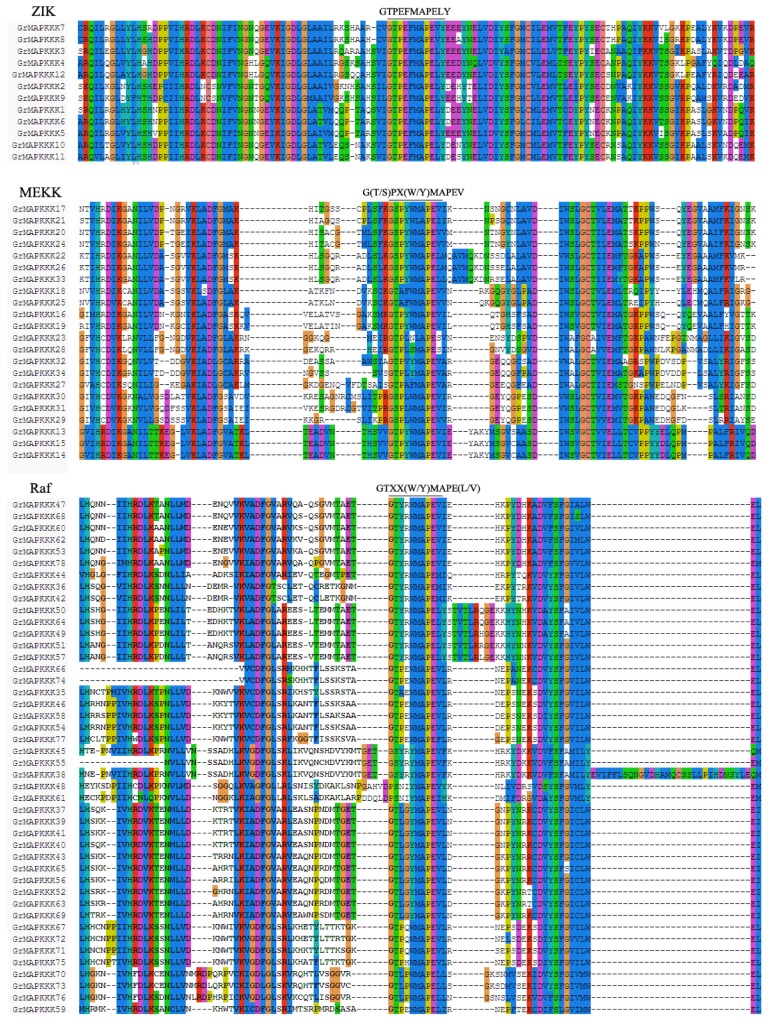
Protein sequence alignment of MAPKKK genes in *G. raimondii*. Alignment was performed using ClustalW program. The highlighted part shows the conserved signature motif.

Conserved domains in the GrMAPKKK protein sequences were analyzed using the PROSITE program. The characteristic feature of the ZIK family is the conserved signature, GTPEFMAPELY (Figure 4). The presence of this specific signature in a protein strongly suggests that it is a member of the ZIK family. In addition, a majority of the family members were found to have a *N*-terminal kinase domain, which was consistent with their orthologs in Arabidopsis, rice and maize. The MEKK family in plants has been shown to be similar to animal MEKKs and yeast MAPKKKs. Members of this family have less conserved protein structures and a shared motif: G(T/S)PX(W/F)MAPEV. Their kinase domain is located either at the *N* or *C*-terminal or in the central part of sequences. In contrast to the ZIK family, most of the Raf family proteins have a *C*-terminal kinase domain and a long *N*-terminal regulatory domain. GTXX(W/Y)MAPE(L/V) was a less conserved signature in these subfamily members. The great diversity in MAPKKKs may allow them to regulate many specific signaling pathways in plants, despite the relatively limited numbers of MAPKKs and MAPKs.

### 2.4. *In Silico* Analysis of Expression of MAPKKKs Based on Transcriptome Sequencing Data

Transcriptome analysis was previously used for identification of protein-coding genes during genome annotation of *G. raimondii* [27]. In this study, sequenced reads that were mapped on the MAPKKK sequences were converted to RPKM to estimate gene expression levels. The data was downloaded from the NCBI and the search was performed using at least 20 nucleotide long signatures.

**Figure 5:**
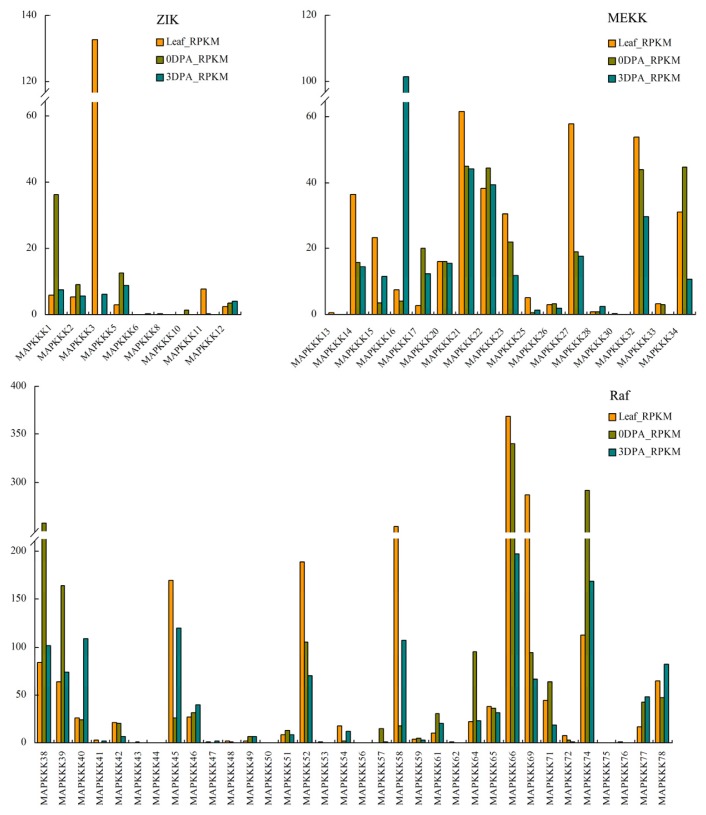
Transcript abundance of MAPKKK genes in mature leaves, 0-DPA ovules and 3-DPA ovules of *G. raimondii*. The transcript expressions were calculated using RPKM method.

Transcript abundance was examined in mature leaves, 0-DPA ovules and 3-DPA ovules. Our analysis revealed that 60 MAPKKK genes (76.92%) were expressed (Figure 5). Some family members exhibited tissue-specific expression, whereas others were more ubiquitously expressed. The expression patterns of some duplicate genes were partially redundant, which suggested that sub-functionalization had occurred during their evolution. Among the ZIK family members, *GrMAPKKK1* was the most highly expressed member in 0-DPA ovules. It is an ortholog of At5g41990, which is called *AtWNK kinase 8* (*WNK8*) in Arabidopsis. AtWNK8 kinase can phosphorylate AtRGS1 (regulator of G-protein signaling 1) and causes AtRGS1 endocytosis, which is required for both G-protein-mediated sugar signaling and cell proliferation [32]. Wang *et al.* [33] showed that the T-DNA knockout *AtWNK8* mutation caused early flowering in Arabidopsis. *GrMAPKKK3* was the most highly expressed member in mature leaves. It was also expressed in 3-DPA ovules, but not in 0-DPA ovules. *GrMAPKKK16* is a member of MEKK subfamily and is an ortholog of *ANP1*/*AtMEKK1* from Arabidopsis. It was most highly expressed in 3-DPA ovules. Previous studies demonstrated that Arabidopsis *ANP1* was functionally redundant to *ANP2* and *ANP3* in the positive regulation of cytokinesis [34]. Two of the three double-mutant combinations displayed defects in cell division and growth. The mutants cause aberrant development of roots, shoots, cotyledons, rosette and cauline leaves, where many cells with incomplete daughter walls are seen [35,36]. In the Raf family, 11 MAPKKK genes (*GrMAPKKK38*, *GrMAPKKK39*, *GrMAPKKK42*, *GrMAPKKK52*, *GrMAPKKK57*, *GrMAPKKK61*, *GrMAPKKK64*, *GrMAPKKK66*, *GrMAPKKK69*, *GrMAPKKK71* and *GrMAPKKK74*) were more highly expressed in 0-DPA ovules than in 3-DPA ovules, whereas 8 MAPKKK genes (*GrMAPKKK40*, *GrMAPKKK45*, *GrMAPKKK46*, *GrMAPKKK47*, *GrMAPKKK54*, *GrMAPKKK58*, *GrMAPKKK77* and *GrMAPKKK78*) were more highly expressed in 3-DPA ovules than in 0-DPA ovules. *GrMAPKKK46*, *GrMAPKKK54* and *GrMAPKKK58* had a common ortholog, *CTR1* in Arabidopsis. *CTR1* was the first discovered component of the ethylene signal transduction pathway. It acts as a negative regulator of ethylene signaling because the *ctr1* mutant alleles exhibited a constitutive ethylene response phenotype [37]. In the absence of ethylene, *CTR1* interacts with ethylene receptors, thereby actively suppressing the ethylene signal response [37,38]. After the binding of ethylene to the receptors, *CTR1* becomes inactivated and the signaling cascade is initiated. Ethylene governs a range of developmental and response processes in plants. After undertaking genomic, genetic, molecular biological and physiological studies, Shi *et al.* [39] demonstrated that ethylene had a prominent role in promoting cotton fiber cell elongation. Exogenously applied ethylene could promote robust fiber cell expansion, whereas its biosynthetic inhibitor specifically suppressed fiber growth. The higher expression of the three *CTR1* homologs: *GrMAPKKK46*, *GrMAPKKK54* and *GrMAPKKK58* in no-fiber *G. raimondii*, suggested that they had a negative effect on early fiber cell development.

## 3. Experimental Section

### 3.1. Sequences Data and Database Search

Multiple database searches were performed to identify the MAPKKK genes in *G. raimondii.* The completed genome sequence of this species was downloaded from the National Centre for Biotechnology Information (NCBI) [40]. Its BioProject accession is PRJNA171262. The protein sequences were obtained from Cotton Genome Project (CGP) and were used to construct a local protein database [41]. It comprised 40,976 sequences. The method used to identify the GrMAPKKK genes was similar to that used for rice and maize [24,25]. The MAPKKK proteins from Arabidopsis and rice were used as query sequences. They were collected from the published literature and downloaded from The Arabidopsis Information Resource (TAIR) and the Rice Genome Annotation Project, respectively [42,43]. The BLAST search was carried out using BLASTP. The aligned parts were inspected and compared manually in order to determine their identity. The *G. raimondii* proteins that had a 50% identity with the query sequence were selected out. These proteins were aligned with themselves and any redundancy removed. Sequence characterizations of the remaining proteins, including the serine/threonine protein kinase active site, the protein kinase domain and the protein kinases ATP-binding region were checked. Motif scanning was done using the PROSITE program at ExPASy [44].

### 3.2. pI, Mw and Subcellular Localization Predictions for MAPKKKs

The theoretical pI and *M*w of the proteins were calculated by the Compute pI/*M*w tool in the ExPASy server [45]. Protein pI was calculated using the *pK* values of amino acids, as described by Bjellqvist *et al.* [46], which were defined by examining polypeptide migration between pH 4.5 and 7.3 in an immobilized pH gradient gel environment with 9.2 M and 9.8 M urea at 15 °C or 25 °C. Protein *Mw* was calculated by adding the average isotopic masses of the amino acids in the protein and the average isotopic mass of one water molecule. Molecular weight values are given in Daltons (Da) and the subcellular localization of each MAPKKK was analyzed using the CELLO v2.5 server [47].

### 3.3. Chromosomal Locations and Gene Structure Analysis

The cDNA sequences of *G. raimondii* were obtained from the Cotton Genome Project. To determine the locations of the MAPKKK genes on chromosomes, their sequences were used as query sequences for a BLASTN search against the whole *G. raimondii* genome. The exon/intron structures for individual MAPKKK genes were determined by aligning the cDNA sequences to their corresponding genomic DNA sequences.

### 3.4. Phylogenetic Analysis and Gene Duplication of MAPKKK Genes

The full-length protein sequences of the MAPKKKs were multi-aligned using the ClustalW2 program [48]. Phylogenetic trees were constructed by employing the NJ method and the ME method found in the MEGA v5 software suite. For both methods, bootstrap testing of the phylogeny was performed with 1000 replications. Other parameters followed the default parameters. The TREEVIEW program and MEGA v5 were used to display the phylogenetic trees. The following criteria were used to define the gene duplication: (1) the alignment length covered > 80% of the longer gene; (2) the aligned region had an identity > 80% and (3) only one duplication event was counted for tightly linked genes.

### 3.5. Expression Analyses of the MAPKKK Genes

The expression pattern of the MAPKKK genes was analyzed in three tissue samples: mature leaves, 0-DPA ovules and 3-DPA ovules of *G. raimondii*. Transcriptome sequencing data for these three samples were obtained from the NCBI Sequence Read Archive (SRA). The accession numbers were: SRX111367, SRX111365 and SRX111366, respectively. Sequenced reads that were mapped on the MAPKKK sequences were converted to RPKM in order to estimate gene expression levels [49]. The formula used was:

$$\text{RPKM}=10^{6}C/(NL/10^{3})$$

where *C* is the number of reads that were uniquely aligned to the transcript, *N* is the total number of reads that were uniquely aligned to all the transcripts in a specific sample and *L* is number of bases in the transcript.

## 4. Conclusions

MAPK cascades act as important signal transduction modules in eukaryotes for many different cellular activities. A MAPK cascade, in its simplest form, consists of a MAPKKK-MAPKK-MAPK module that is linked in various ways to upstream receptors and downstream targets. As the first level of the phosphorylating cascade, the MAPKKK family has the most members and exhibits the most divergence. So far, 80, 75 and 74 MAPKKK genes in the Arabidopsis, rice and maize genomes have been reported, respectively. In this study, we identified 78 MAPKKK genes in the sequenced genome of diploid cotton *G. raimondii*. Their phylogenetic relationship, genomic distribution, conserved protein motif and exon/intron organization were characterized. The MAPKKK genes were divided into three major groups. There were 12 MAPKKKs in the ZIK group, 22 MAPKKKs in the MEKK group and 44 MAPKKKs in the Raf group. The genes in each group had a similar motif in the deduced amino acid sequences, which supported their identification as members of ZIK, MEKK and Raf. The ZIK and MEKK genes were located on chromosomes: II, III, IV, V, VI, VII, VIII, XI, XII and XIII. The Raf genes were distributed across all 13 chromosomes. Although there were numerous MAPKKKs paralogs displaying high levels of sequence similarity, few clusters containing closely related genes were found. This pattern probably reflects a series of whole genome, chromosomal and large segmental duplication events for the *G. raimondii* genome. Based on transcriptome sequencing data, we analyzed expression patterns of MAPKKKs at the fiber initiation stage and in mature leaves. They showed dramatic differences in expression levels in different tissues. Interestingly, *GrMAPKKK46*, *GrMAPKKK54* and *GrMAPKKK58* had higher expression levels in 3-DPA ovules than in 0-DPA ovules. Their direct ortholog was *CTR1*, which is known to be a negative regulator of the ethylene signaling pathway in Arabidopsis. When combined with the prominent role for ethylene in promoting cotton fiber development, this observation might help us to investigate the regulation mechanism for fiber cell elongation in cotton species. Taken together, our results should facilitate the functional annotation of these first signaling components in the MAPK cascade. Additionally, the results should provide an important foundation for the study of very poorly characterized MAPKKKs in non-sequenced tetraploid cotton.

## Figures and Tables

**Table 1:** Characteristics of MAPK kinase kinase (MAPKKKs) from *G. raimondii*.

Gene name	Accession number	AA	pI	*M*w	Predicted subcellular localization
GrMAPKKK1	Cotton_D_gene_10030976	705	5.24	79.82	Nuclear
GrMAPKKK2	Cotton_D_gene_10024942	294	5.37	33.68	Nuclear
GrMAPKKK3	Cotton_D_gene_10021384	613	5.09	69.23	Nuclear
GrMAPKKK4	Cotton_D_gene_10025360	592	6.19	67.33	Nuclear
GrMAPKKK5	Cotton_D_gene_10025689	668	4.96	75.59	Nuclear
GrMAPKKK6	Cotton_D_gene_10016819	686	5.17	77.71	Nuclear
GrMAPKKK7	Cotton_D_gene_10011135	734	4.99	83.01	Nuclear
GrMAPKKK8	Cotton_D_gene_10007538	727	5.47	83.62	Nuclear
GrMAPKKK9	Cotton_D_gene_10006903	299	5.21	34.12	Nuclear
GrMAPKKK10	Cotton_D_gene_10028727	643	5.56	72.19	Nuclear
GrMAPKKK11	Cotton_D_gene_10019741	609	5.25	67.84	Nuclear
GrMAPKKK12	Cotton_D_gene_10036331	593	5.36	67.67	Nuclear
GrMAPKKK13	Cotton_D_gene_10018040	1370	5.96	150.94	Nuclear
GrMAPKKK14	Cotton_D_gene_10034692	1399	5.86	154.46	Nuclear
GrMAPKKK15	Cotton_D_gene_10029669	1397	5.94	153.50	Nuclear
GrMAPKKK16	Cotton_D_gene_10017021	662	5.79	72.69	Nuclear
GrMAPKKK17	Cotton_D_gene_10001555	897	9.22	96.45	Nuclear
GrMAPKKK18	Cotton_D_gene_10002230	577	5.41	64.07	Nuclear
GrMAPKKK19	Cotton_D_gene_10003410	643	6.06	70.78	Nuclear
GrMAPKKK20	Cotton_D_gene_10019751	661	9.20	71.56	Nuclear
GrMAPKKK21	Cotton_D_gene_10030510	896	9.29	96.69	Nuclear
GrMAPKKK22	Cotton_D_gene_10032983	711	8.99	78.75	Nuclear
GrMAPKKK23	Cotton_D_gene_10008602	338	4.97	37.35	Cytoplasmic
GrMAPKKK24	Cotton_D_gene_10038046	660	9.05	71.32	Nuclear
GrMAPKKK25	Cotton_D_gene_10039321	576	5.63	63.66	Nuclear
GrMAPKKK26	Cotton_D_gene_10040437	742	8.68	81.55	Nuclear
GrMAPKKK27	Cotton_D_gene_10024896	437	4.65	48.84	Cytoplasmic
GrMAPKKK28	Cotton_D_gene_10030314	336	5.78	36.99	Cytoplasmic
GrMAPKKK29	Cotton_D_gene_10000305	466	7.85	52.12	Chloroplast
GrMAPKKK30	Cotton_D_gene_10025330	443	5.51	49.41	Nuclear
GrMAPKKK31	Cotton_D_gene_10006972	495	5.63	55.42	Cytoplasmic
GrMAPKKK32	Cotton_D_gene_10033856	438	5.49	48.15	Nuclear
GrMAPKKK33	Cotton_D_gene_10030328	613	8.47	67.53	Nuclear
GrMAPKKK34	Cotton_D_gene_10031221	446	5.58	49.38	Cytoplasmic
GrMAPKKK35	Cotton_D_gene_10040125	986	5.25	108.05	Nuclear
GrMAPKKK36	Cotton_D_gene_10035121	371	9.14	42.42	Nuclear
GrMAPKKK37	Cotton_D_gene_10027896	374	7.92	41.92	Nuclear
GrMAPKKK38	Cotton_D_gene_10037878	460	8.63	51.88	Mitochondrial
GrMAPKKK39	Cotton_D_gene_10019447	381	7.92	42.44	Nuclear
GrMAPKKK40	Cotton_D_gene_10030570	399	5.42	44.40	Nuclear
GrMAPKKK41	Cotton_D_gene_10032760	377	7.94	42.04	Nuclear
GrMAPKKK42	Cotton_D_gene_10031200	374	8.92	42.57	Nuclear
GrMAPKKK43	Cotton_D_gene_10038072	420	7.89	46.91	Cytoplasmic
GrMAPKKK44	Cotton_D_gene_10012774	415	8.11	46.35	Chloroplast
GrMAPKKK45	Cotton_D_gene_10014886	427	8.62	48.02	Mitochondrial
GrMAPKKK46	Cotton_D_gene_10028168	849	6.13	94.22	Nuclear
GrMAPKKK47	Cotton_D_gene_10011865	609	5.85	68.46	Cytoplasmic
GrMAPKKK48	Cotton_D_gene_10000379	540	9.03	61.20	Mitochondrial
GrMAPKKK49	Cotton_D_gene_10010262	351	8.09	39.70	Cytoplasmic
GrMAPKKK50	Cotton_D_gene_10019394	354	6.57	39.98	Cytoplasmic
GrMAPKKK51	Cotton_D_gene_10002587	353	8.92	39.42	Cytoplasmic
GrMAPKKK52	Cotton_D_gene_10035493	391	8.38	43.63	Cytoplasmic
GrMAPKKK53	Cotton_D_gene_10025598	563	5.91	63.78	Cytoplasmic
GrMAPKKK54	Cotton_D_gene_10013051	857	6.18	94.66	Nuclear
GrMAPKKK55	Cotton_D_gene_10033568	402	8.42	44.93	Mitochondrial
GrMAPKKK56	Cotton_D_gene_10035982	391	8.35	43.59	Nuclear
GrMAPKKK57	Cotton_D_gene_10035556	355	8.82	39.84	Cytoplasmic
GrMAPKKK58	Cotton_D_gene_10003920	860	6.49	95.59	Nuclear
GrMAPKKK60	Cotton_D_gene_10005866	575	5.71	65.08	Cytoplasmic
GrMAPKKK61	Cotton_D_gene_10023083	477	9.15	54.17	Mitochondrial
GrMAPKKK62	Cotton_D_gene_10018183	552	6.09	62.28	Cytoplasmic
GrMAPKKK63	Cotton_D_gene_10031629	391	8.65	43.44	Nuclear
GrMAPKKK64	Cotton_D_gene_10019101	352	5.98	39.79	Cytoplasmic
GrMAPKKK65	Cotton_D_gene_10003024	381	7.05	42.54	Cytoplasmic
GrMAPKKK66	Cotton_D_gene_10015094	904	5.45	100.46	Cytoplasmic
GrMAPKKK67	Cotton_D_gene_10021266	782	6.87	86.35	Nuclear
GrMAPKKK68	Cotton_D_gene_10034448	576	6.26	65.42	Nuclear
GrMAPKKK69	Cotton_D_gene_10005280	383	8.19	42.40	Cytoplasmic
GrMAPKKK70	Cotton_D_gene_10028280	1137	5.56	126.21	Nuclear
GrMAPKKK71	Cotton_D_gene_10015701	848	5.88	94.81	Nuclear
GrMAPKKK72	Cotton_D_gene_10012401	765	6.54	84.93	Nuclear
GrMAPKKK73	Cotton_D_gene_10016031	1107	5.46	122.84	Cytoplasmic
GrMAPKKK74	Cotton_D_gene_10037238	854	5.18	94.24	Cytoplasmic
GrMAPKKK75	Cotton_D_gene_10005797	743	8.17	82.52	Nuclear
GrMAPKKK76	Cotton_D_gene_10004434	1360	5.18	147.72	Nuclear
GrMAPKKK77	Cotton_D_gene_10011720	879	6.80	97.86	Nuclear
GrMAPKKK78	Cotton_D_gene_10033954	535	6.43	60.56	Cytoplasmic

AA: Amino acid; pI: The theoretical isoelectric point of proteins; *M*w: The theoretical molecular weight of proteins.
